# Impact of genetic polymorphisms on the response to transdermal fentanyl in cancer patients—a single-center, prospective, biological study

**DOI:** 10.3389/fpain.2026.1834786

**Published:** 2026-08-17

**Authors:** Lucia Angelini, Irene Azzali, Andrea Carlini, Vincenza Andrisano, Serena Montanari, Monia Dall'agata, Giorgia Simonetti, Maria Teresa Bochicchio, Matteo Paganelli, Vanessa Valenti, Paola Cravero, Margherita Currà, Linda Petrini, Costanza Maria Donati, Alessio G. Morganti, Andrea Ardizzoni, Oscar Edoardo Corli, Marco Cesare Maltoni, Romina Rossi, Marianna Ricci

**Affiliations:** 1 Palliative Care Unit, IRCCS "Istituto Romagnolo Per Lo Studio Dei Tumori (IRST), Dino Amadori", Meldola, FC, Italy; 2 Unit of Biostatistics and Clinical Trials, IRCCS Istituto Romagnolo per lo Studio DeiTumori (IRST) "Dino Amadori", Meldola, Italy; 3 Department of Medical and Surgical Sciences (DIMEC), Alma Mater Studiorum-University of Bologna, Bologna, Italy; 4 Department for Life Quality Studies, University of Bologna, Rimini, Italy; 5 Biosciences Laboratory, Istituto di Ricovero e Cura a Carattere Scientifico IstitutoRomagnolo per lo Studio dei Tumori "Dino Amadori,", Meldola, Italy; 6 Radiation Oncology, IRCCS Azienda Ospedaliero-Universitaria di Bologna, Bologna, Italy; 7 Medical Oncology, IRCCS Azienda Ospedaliero-Universitaria di Bologna, Bologna, Italy; 8 Istituto di Ricerche Farmacologiche Mario Negri-IRCCS, Milano, Italy

**Keywords:** cancer pain, opioid response, pharmacogenetics, pharmacokinetics, transdermal fentanyl

## Abstract

**Background/objectives:**

Transdermal fentanyl (TDF) is widely used for moderate-to-severe cancer pain, yet substantial variability in fentanyl exposure, analgesic response, and tolerability remains incompletely explained by clinical factors. We evaluated whether pharmacogenetic variability is associated with pharmacokinetic parameters and clinical outcomes in cancer patients receiving TDF.

**Methods:**

In this prospective, single-center study, adults with solid or hematological malignancies and chronic cancer pain treated with stable-dose 72 h TDF were enrolled. Blood samples were collected for pharmacokinetic and pharmacogenetic analyses. Serum fentanyl concentrations were quantified using validated gas chromatography–mass spectrometry, and AUC, C_max_, and t_max_ were calculated using non-compartmental analysis. Genotyping was performed using a predefined pharmacogenetic panel. Analgesic response was defined as a ≥2-point reduction in Numeric Rating Scale score or score = 0 at 72 h. Statistical analyses included correction for multiple testing.

**Results:**

49 patients were included (median age 65 years; 98% stage IV disease); 38 (77.5%) were responders. No significant differences in AUC, C_max_, or t_max_ were observed between responders and non-responders. TPMT and UGT2B7 variants showed unadjusted associations with clinical response and pharmacokinetic parameters, respectively; however, none remained statistically significant after correction for multiple testing. No significant associations were observed for CYP3A4/5 or ABCB1 variants, nor between genetic variants and adverse events. Pain reduction correlated with improved functional outcomes.

**Conclusions:**

In this exploratory cohort, pharmacogenetic variability was not robustly associated with TDF pharmacokinetics, analgesic response, or tolerability after correction for multiple testing. These findings do not support the current clinical implementation of pharmacogenetic-guided TDF therapy, but provide hypothesis-generating signals for future studies.

## Introduction

Pain is a highly prevalent symptom among cancer patients, with estimated rates ranging from 33% in individuals post-curative treatment, to 59% in those receiving active anticancer therapy, and up to 64% in patients with metastatic, advanced, or terminal disease (1). Despite substantial advancements in palliative care, cancer pain management remains a significant clinical challenge.

Transdermal fentanyl (TDF) is one of the most prescribed opioids worldwide and is recommended for patients with moderate to severe cancer pain on stable opioid therapy (2). Its transdermal delivery system provides continuous systemic drug diffusion over 72 h, ensuring sustained analgesia in chronic pain settings (3). Fentanyl is a potent synthetic μ-opioid receptor agonist, approximately 75–100 times more potent than morphine (4). After absorption, it crosses the blood–brain barrier partly through P-glycoprotein (ABCB1) transport and exerts its pharmacological effects predominantly via μ-opioid receptor activation, with low affinity for *δ*- and *κ*-opioid receptors (5). It is mainly metabolized by hepatic cytochrome P450 enzymes CYP3A4 and CYP3A5 via oxidative N-dealkylation to inactive metabolites, with approximately 10% excreted unchanged in urine (6, 7). Therapeutic serum concentrations are typically reached after 12–16 h, with maximum concentrations (C_max_) occurring at 36 h (time to reach maximum concentration, t_max_). Depending on the dose, plasma concentrations range from 0.3 ng/mL for a 12.5 μg/hour patch to 2.6 ng/mL for a 100 μg/hour patch (8).

Clinical factors such as age, sex, organ function, body temperature, muscle wasting, cachexia and dehydration have been proposed as contributors to inter- and intraindividual variability in TDF response, safety and pharmacokinetics (PK) (9–12). Nevertheless, these variables explain only a limited proportion of the observed heterogeneity, suggesting the involvement of additional determinants, including the pharmacogenetic profile. Several studies have investigated genetic variants affecting fentanyl metabolism, particularly CYP3A4 and CYP3A5. A Japanese study found that homozygous carriers of a CYP3A5 variant had faster fentanyl absorption and an increased incidence of central adverse events (AEs) (13). In contrast, analysis of CYP3A5*3 and CYP3A4*22 variants in a large cohort of 620 cancer patients receiving TDF indicated that these polymorphisms accounted for only a small fraction of serum fentanyl variability (14). Studies of ABCB1 polymorphisms in patients with chronic cancer pain have shown that homozygous variants correlate with improved pain control and a reduced need for rescue medications (13). Other pharmacogenetic studies have focused on OPRM1 (μ-opioid receptor 1), COMT (catechol-O-methyltransferase), NAT2 (N-acetyltransferase 2) and UGT2B7 (UDP-glucuronosyltransferase-2B7) polymorphisms and their potential associations with opioid response and toxicity in cancer patients, with overall inconsistent or inconclusive findings (15–17). Thus, the extent to which the pharmacogenetic profile meaningfully influence TDF PK, analgesic response, and tolerability remains uncertain.

Taken together, these findings underscore the need for further exploration of pharmacogenetic determinants of fentanyl response in cancer patients. The primary objective of this study was to investigate whether pharmacogenetic variability may be associated with differences in clinical response in cancer patients receiving TDF. Secondary aims included examining potential associations between selected genetic variants and fentanyl pharmacokinetic parameters, dose requirements, and opioid-related AEs.

## Materials and methods

This biological, prospective, single-center clinical study was conducted at the Istituto Romagnolo per lo Studio dei Tumori (IRST) “Dino Amadori” between September 2018 and September 2021. Adult patients with solid or hematological malignancies experiencing chronic cancer pain and treated with a 72 h TDF patch at stable doses were eligible. Inclusion criteria were: age ≥ 18 years; confirmed diagnosis of solid or hematological malignancy; treatment with TDF for chronic cancer pain; adequate hepatic function; absence of major psychiatric disorders; and ability to reliably report pain intensity over time. Exclusion criteria included: pregnancy or breastfeeding, history of substance abuse or excessive alcohol consumption, known hypersensitivity to study drugs, and concomitant use of medications with clinically relevant interactions with fentanyl. TDF dosing was maintained unchanged throughout the study period. Breakthrough pain and AEs were managed according to standard clinical practice. All patients received verbal and written information and provided written informed consent. The study was conducted in accordance with Italian regulations governing biomedical research and was approved by the local Ethics Committee.

Peripheral blood samples (3 mL; EDTA tubes, two tubes at first collection only) were collected at predefined time points for pharmacokinetic and pharmacogenetic analyses: at TDF patch application (T0), and at 6 h (T1), 18 h (T2), 48 h (T3), and 72 h (T4) post-application. All patients were receiving stable-dose TDF at enrolment; therefore, T0 corresponded to scheduled patch application or replacement during ongoing stable treatment rather than first-ever exposure to fentanyl. Pain intensity was assessed using the Numeric Rating Scale (NRS) at T0 and T4. The Brief Pain Inventory (BPI) questionnaire was administered at each time point to explore pain-related functional interference, including general activity, mood, walking ability, normal work, relations with others, sleep, and enjoyment of life. Patients were classified as responders if they achieved an absolute reduction of at least 2 points in NRS score between T0 and T4 or an NRS score of 0 at T4; all others were classified as non-responders. This threshold was selected because an approximately 2-point reduction on a 0–10 pain intensity scale is commonly considered clinically meaningful in pain research. BPI scores were analyzed as secondary multidimensional outcomes. Clinical data collected included age, sex, tumor type and stage, body mass index (BMI), Eastern Cooperative Oncology Group performance status (ECOG PS), hydration status, concomitant medications, fentanyl dose, AEs, and opioid use for breakthrough pain.

Serum fentanyl concentrations were analyzed at the Department of Life Quality Studies, Rimini Campus, University of Bologna, using a previously validated and quality-controlled gas chromatography–mass spectrometry method with established sensitivity, specificity, and analytical precision (18). Pharmacokinetic parameters were calculated using non-compartmental analysis. Area under the concentration–time curve (AUC) was estimated using the trapezoidal rule, while C_max_ and t_max_ were derived directly from observed concentration–time data (19).

Genomic DNA was extracted from peripheral whole blood using the Maxwell® RSC Whole Blood DNA Kit (Promega) and genotyped with the Axiom™ PharmacoFocus Assay (Thermo Fisher Scientific). This assay interrogates approximately 2,000 variants across 150 predefined pharmacogenes involved in drug metabolism, transport, and pharmacodynamics, including genes encoding drug-metabolizing enzymes (e.g., CYP family), transporters (e.g., ABCB1), opioid receptors (e.g., OPRM1), and conjugating enzymes (e.g., UGT2B7 and NAT2). Genes of primary pharmacological interest were selected *a priori* based on their known or plausible involvement in fentanyl metabolism, transport, or opioid pharmacodynamics, including CYP3A4, CYP3A5, ABCB1, OPRM1, COMT, UGT2B7, and NAT2. Other pharmacogenes captured by the assay, including genes not directly related to fentanyl metabolism such as thiopurine S-methyltransferase (TPMT) and vitamin K epoxide reductase complex subunit 1 (VKORC1), were analyzed exploratorily as part of the broader predefined panel. Genotypes were translated into predicted functional phenotypes according to standardized allele-function classifications. Metabolic enzymes were categorized as ultrarapid, rapid, normal, intermediate, or poor metabolizers, transporters as increased, normal, decreased, or poor function. Gene-specific classifications were applied where appropriate [e.g., acetylation status for NAT genes; resistant/sensitive phenotypes for VKORC1/CYP2C; deficient status for glucose-6-phosphate dehydrogenase (G6PD)]. For analytical purposes, patients were grouped into normal-function or altered-function categories based on predicted functional impact. The normal-function group included normal metabolizers and favorable phenotypes, whereas the altered-function group comprised intermediate/poor metabolizers and other reduced, increased, resistant, sensitive, or unfavorable functional phenotypes. Further methodological details are provided in Text S1 and Supplementary Table S1.

Descriptive statistics were reported as frequencies and percentages for categorical variables and means with standard deviations or medians with interquartile ranges (IQR) for continuous variables. Associations between PK, pharmacogenetic profiles, clinical characteristics, and clinical response were assessed using Fisher's exact test or Chi-squared test for categorical variables and the Wilcoxon rank-sum test for continuous variables. To account for multiple comparisons in genetic analyses, *p*-values were adjusted using the Benjamini-Hochberg procedure across all pharmacogenetic comparisons. An adjusted *p*-value < 0.05 was considered statistically significant. Given the exploratory nature of the study and the limited sample size, both unadjusted and adjusted *p*-values are reported. All statistical analyses were performed using STATA version 15.0 (StataCorp, College Station, TX, USA) and R Studio.

## Results

### Demographic characteristics

A total of 49 patients (23 females and 26 males) were included, with a median age of 65 years (range 32–83). Most patients had a normal BMI (63.3%) and an ECOG PS ≥ 2 (77.6%). Nearly all patients (98%) had stage IV disease. The most frequent tumor types were gastrointestinal malignancies (30.6%) and multiple myeloma (12.2%). TDF doses ranged from 12 to 150 µg/h, with most patients receiving 25–50 µg/h. Concomitant medications recorded at baseline are reported in Supplementary Table S2. AEs were reported in 32.7% of cases. According to predefined criteria, 38 patients (77.5%) were classified as responders. The distribution of NRS scores at baseline and at T4 is shown in Supplementary Figure S1. Among clinical variables, sex showed an unadjusted association with treatment response (*p* = 0.05), with a higher proportion of responders among males; however, this association did not remain statistically significant (adjusted *p* = 0.50). Detailed demographic and clinical characteristics are summarized in Table 1.

**Table 1:** Demographic characteristics.

Characteristics	Overall (*n* = 49)	Responders (*n* = 38)	Non-responders (*n* = 11)	*p*
Sex—no. (%)				
Female	23 (47)	15 (39)	8 (74)	
Male	26 (53)	23 (61)	3 (26)	0.05
Age at first treatment—yr				
Median	65	64	67	
Range	(32–83)	(32–83)	(57–82)	0.32
BMI—no. (%)				
Underweight (<18.5)	6 (12)	3 (8%)	3 (26%)	
Normal range (18.5–24.9)	31 (63)	25 (66%)	6 (56%)	
Overweight (25–29.9)	8 (16)	6 (16%)	2 (18%)	
Obese (≥30)	4 (8)	4 (10%)	0	0.26
ECOG performace status—no. (%)				
1	11 (22)	9 (24%)	2 (18%)	
2	20 (41)	15 (39%)	5 (45%)	
3	18 (37)	14 (37%)	4 (36%)	0.91
Stage—no. (%)				
III	1 (2)	1 (3)	0	
IV	48 (98)	36 (97)	11 (100)	1
Fentanyl dosage µg/h—no. (%)				
12	8 (16)	5 (13)	3 (26)	
25	15 (31)	13 (34)	2 (18)	
50	14 (29)	10 (26)	4 (36)	
75	6 (12)	5 (13)	1 (10)	
100	4 (8)	3 (8)	1 (10)	
150	2 (4)	2 (6)	0	0.74
Side effects—no (%)				
Yes	16 (33)	12 (32)	4 (36)	
No	30 (61)	23 (61)	7 (64)	
Missing	3 (6)	3 (5)	0	0.90
Time on Fentanyl treatment—no (%)				
≤30 days	20 (41)	17 (45)	3 (27)	
>30 days	25 (51)	19 (50)	6 (54)	
Missing	4 (8)	2 (5)	2 (19)	0.71
Daily rescue doses—no (%)				
≤2	40 (82)	33 (87)	7 (64)	
>2	9 (8)	5 (13)	4 (36)	0.18

BMI, Body Mass Index; ECOG PS, Eastern Cooperative Oncology Group performance status.

### Pharmacogenetic profile and clinical and pharmacokinetic outcomes

No statistically significant differences in pharmacokinetic parameters (AUC, C_max_, t_max_) were observed between responders and non-responders. Among all genes analyzed, TPMT phenotype showed an unadjusted association with treatment response (*p* = 0.009), as all individuals with altered-function phenotypes were non-responders. However, this association did not remain statistically significant after correction for multiple testing. No other gene showed a meaningful association with clinical response (Table 2, Supplementary Tables S3, S4). Stratification by fentanyl dosage (≤50 µg/h vs. >50 µg/h) revealed no significant associations between genotype and dose category (Supplementary Table S5). Similarly, no polymorphisms were significantly associated with the occurrence of AEs and the frequency of AEs did not differ between responders and non-responders (*p* = 0.58) (Supplementary Table S6). Regarding PK, UGT2B7 altered-function phenotypes showed unadjusted associations with higher AUC (*p* = 0.02) and Cmax (*p* = 0.006) compared with normal-function phenotypes. However, these associations did not remain statistically significant after correction for multiple testing. Other genes, including TPMT, CYP3A4, CYP3A5, COMT, and nudix hydrolase 15 (NUDT15), showed *p*-values below 0.1 for at least one pharmacokinetic parameter. However, none of these associations did not remain statistically significant after correction for multiple testing (Tables 3, Supplementary Table S7). Sensitivity analyses using ≥30% and ≥50% reductions in NRS score as alternative definitions of analgesic response yielded results consistent with the primary analysis, with no statistically significant associations between the investigated genetic polymorphisms and response status or pharmacokinetic parameters (Supplementary Tables S8a–c). Sensitivity analyses excluding patients with hematological malignancies (*n* = 6) yielded results consistent with those of the overall cohort, with no relevant changes in the main pharmacokinetic or pharmacogenetic associations (Supplementary Tables S9a–c).

**Table 2:** Genetic polymorphisms and clinical response. The table reports genes with unadjusted *p*-values < 0.3.

Gene phenotype—no (%)	Responders (*n* = 38)	Non-responders (*n* = 11)	*p*	*p-*adj
CYP2CRS12777823				
Normal function	26 (68)	10 (91)		
Altered function	12 (32)	1 (9)	0.25	1
CYP2C8				
Normal function	23 (64)	9 (90)		
Altered function	13 (36)	1 (10)		
Missing	2	1	0.14	1
CYP2C9				
Normal function	23 (62)	9 (82)		
Altered function	14 (38)	2 (18)		
Missing	1	0	0.29	1
TPMT				
Normal function	38 (100)	8 (73)		
Altered function	0	3 (27)	**0** **.** **009**	0.22

CYP, cytochrome P450 family; TPMT, thiopurine S-methyltransferase.

Bold values indicate statistically significant *p*-values (*p* < 0.05).

**Table 3:** Genetic polymorphisms and pharmacokinetics. The table includes only genes for which at least one unadjusted *p*-value was below 0.2 in the comparison of AUC, C_max_, or t_max_ between phenotype groups.

Gene phenotype	AUC			t_max_			C_max_		
	median, IQ-IIIQ	*p*	*p-*adj	median, IQ-IIIQ	*p*	*p-*adj	median, IQ-IIIQ	*p*	*p-*adj
COMT									
Normal function	272.54, 90.75–1,193.85			3.00, 2.00–3.00			4.61, 1.96–24.58		
Altered function	134.44, 52.80–326.85	0.11	0.61	3.00, 1.00–4.00	0.88	0.92	4.00, 1.08–11.90	0.19	0.78
CYP3A4									
Normal function	187.06, 68.57–879.05			3.00, 1.00–4.00			4.23, 1.48–17.04		
Altered function	56.91, 54.77–68.47	0.17	0.61	1.00, 1.00–2.00	0.06	0.85	1.08, 0.99–1.51	0.21	0.78
CYP3A5									
Normal function	11.94, 11.94–11.94			4.00, 4.00–4.00			0.83, 0.83–0.83		
Altered function	164.78, 59.96–786.83	0.08	0.61	3.00, 1.00–3.00	0.19	0.92	4.14, 1.43–17.04	0.25	0.78
NUDT15									
Normal function	142.32, 57.46–660.58			3.00, 1.00–3.00			3.33, 1.18–15.69		
Altered function	1,465.55, 1,009.76–1,921.34	0.10	0.61	2.50, 1.75–3.25	0.87	0.92	27.94, 21.43–34.46	0.10	0.78
TPMT									
Normal function	135.70, 56.91–700.69			3.00, 1.00–3.00			2.65, 1.10–16.95		
Altered function	326.85, 291.18–605.99	0.28	0.88	4.00, 3.50–4.00	0.08	0.85	11.90, 8.63–14.55	0.25	0.78
UGT2B7									
Normal function	78.18, 42.35–187.06			3.00, 1.00–4.00			1.51, 0.83–4.14		
Altered function	326.85, 96.60–1,057.86	**0** **.** **02**	0.56	3.00, 1.00–3.50	0.46	0.92	6.70, 1.97–19.80	**0** **.** **006**	0.14

AUC, area under the concentration–time curve; C_max_, maximum (peak) plasma concentration; CYP, cytochrome P450 family; IQ–IIIQ, interquartile range (25th–75th percentile); COMT, catechol-O-methyltransferase; NUDT15, nudix hydrolase 15; TPMT, thiopurine S-methyltransferase; t_max_, time to maximum concentration; UGT2B7, UDP-glucuronosyltransferase-2B7.

Bold values indicate statistically significant *p*-values (*p* < 0.05).

### Pain intensity and quality of life

A moderate positive association was observed between changes in NRS scores and changes in the BPI Total Pain Interference Score from T0 to T4 (Spearman's rho = 0.39, *p* = 0.006), particularly in the work (*p* = 0.008) and relations with other people (*p* = 0.01) domains, as reported in Supplementary Table S10. Furthermore, a statistically significant difference in BPI total score change was detected between responders and non-responders. Responders showed a mean change of −4.29 (95% CI: −8.44 to −0.14), whereas non-responders showed a mean change of 4.80 (95% CI: −5.88 to 15.48; *p* = 0.02), suggesting that pain relief was associated with improvements in daily functioning and quality of life. Exploratory analyses of individual BPI interference domains are reported in Supplementary Table S11. Among individual domains, significant between-group differences were observed for normal work (*p* = 0.008) and relations with other people (*p* = 0.01), while general activity showed a non-statistically significant trend toward improvement (*p* = 0.06).

## Discussion

The European Association for Palliative Care (EAPC) and European Society of Medical Oncology (ESMO) guidelines support the use of TDF as a viable alternative to oral opioids for the management of moderate to severe cancer pain requiring stable opioid administration. This recommendation is particularly relevant for patients experiencing nausea, vomiting, dysphagia and constipation (2, 20). A systematic review comparing TDF with oral opioids has demonstrated comparable analgesic efficacy, with potential advantages of transdermal formulations in terms of constipation and patient preference (21, 22). Nevertheless, fentanyl PK (23), analgesic efficacy, and safety profile (24) are characterized by marked inter- and intraindividual variability. Established contributors include age, sex, organ function, body temperature, cachexia, dehydration, and concomitant use of CYP3A4 modulators (9–11, 14). However, these acquired factors do not fully explain the heterogeneity observed in systemic exposure, clinical efficacy, and tolerability, supporting investigation into pharmacogenetic determinants (25, 26). At the same time, cancer pain should not be conceptualized as a purely pharmacological or pharmacokinetic phenomenon. Pain intensity, functional interference, and perceived opioid response may also be influenced by psychological distress, sleep disturbance, pain catastrophizing, social support, communication patterns, and the broader palliative-care context (27, 28). Therefore, the pharmacogenetic findings of this study should be interpreted within a multidimensional biopsychosocial framework.

In this exploratory study, we investigated the influence of clinical and genetic variables on TDF PK, analgesic response, and tolerability. Among clinical variables, sex showed a borderline unadjusted association with treatment response (*p* = 0.05), with a higher proportion of responders among males; however, this association did not remain significant after correction for multiple clinical comparisons (adjusted *p* = 0.50). This finding should therefore be considered with caution and should not be regarded as evidence of a clinically meaningful sex-related difference in TDF response. Although previous studies have reported sex-related differences in fentanyl PK, potentially reflecting higher CYP3A4 activity in females and differences in body composition (29, 30), our data do not allow firm conclusions regarding the role of sex in analgesic response. Conversely, no significant association was observed between BMI and clinical response.

Our pharmacogenetic analyses identified an unadjusted association between TPMT polymorphisms and clinical response, with patients carrying altered-function phenotypes (IM and PM) more frequently classified as non-responders. This association did not remain significant after correction for multiple testing and should therefore be considered strictly exploratory and hypothesis-generating. TPMT is primarily involved in thiopurine metabolism, and no direct mechanistic link with fentanyl PK has been established (31, 32). Notably, none of the patients in our cohort, including those with altered-function TPMT phenotypes, were receiving thiopurine-based therapy. The observed signal may reflect indirect interactions with concomitant therapies or broader metabolic pathways rather than a direct effect on fentanyl metabolism. Replication in larger cohorts is required before biological or clinical relevance can be inferred. In addition, an altered-function UGT2B7 phenotype was associated with higher AUC and C_max_ values compared with normal-function phenotypes. As with TPMT, this association did not withstand correction for multiple testing. UGT2B7 encodes a UDP-glucuronosyltransferase involved in the conjugation of endogenous compounds and xenobiotics (33). Although fentanyl is primarily metabolized by CYP3A4/5, certain metabolites, including norfentanyl, undergo glucuronidation, providing a plausible—albeit indirect—biological rationale for this finding (17, 34). The rs7439366 variant identified in our cohort has previously been associated with fentanyl sensitivity in surgical settings (35). These observations suggest a potential role of UGT2B7 in modulating fentanyl exposure, but confirmation in adequately powered studies is necessary. In terms of AEs, no genetic polymorphisms were significantly associated with their occurrence, and AEs frequency did not differ between responders and non-responders. This finding is consistent with the result of *Andreassen* et al., who reported that CYP2D6 polymorphisms, affecting a cytochrome P450 enzyme involved in xenobiotic metabolism (36), do not influence oxycodone efficacy in cancer patients (37).

Unlike *Takashina* et al., who reported associations between CYP3A5 polymorphisms, normalized fentanyl concentrations, and central AEs in patients switched from other opioids to TDF, we found no significant associations between CYP3A4/5 phenotype and fentanyl PK, analgesic response, or AEs. These discrepant findings may reflect differences in population, ethnicity, clinical setting, sampling strategy, and analytical methods. In particular, *Takashina* et al. studied Japanese patients undergoing opioid switching from other opioids to TDF, whereas our cohort included predominantly Caucasian patients already receiving stable-dose TDF. Moreover, our study incorporated serial pharmacokinetic sampling across the entire 72 h patch interval, whereas *Takashina* et al. assessed fentanyl concentrations at a single time point. Additional differences in genotype distribution, definitions of clinical outcomes and AEs, analytical methodology, and use of normalized fentanyl concentrations may also have contributed to the divergent findings (13). Conversely, our results are consistent with the large EPOS study by *Barratt* et al., in which CYP3A4*22 and CYP3A5*3 polymorphisms explained only a limited proportion of fentanyl exposure variability after adjustment for clinical factors. Although the EPOS study differed from ours in several respects, including its larger multicenter design, broader patient heterogeneity, single sampling timepoint and analytical methods, both investigations evaluated patients receiving TDF in routine clinical settings. Nevertheless, both studies support the limited standalone predictive value of CYP3A4/5 variants in patients receiving TDF (14). Similarly, no clear association emerged for ABCB1 variants, whose clinical relevance remains uncertain (38). Overall, our findings suggest that single-gene pharmacogenetic markers alone are unlikely to provide sufficient accuracy for individualized TDF dosing without integration of clinical and pharmacokinetic factors.

Changes in NRS scores were positively correlated with changes in BPI pain interference domains, including general activity, work, and interpersonal relationships. A significant difference in total BPI score change was also observed between responders and non-responders, indicating that analgesic response was associated not only with reduced pain intensity but also with improved daily functioning and patient-reported quality of life. The exploratory analysis of individual BPI interference domains further supports the clinical relevance of analgesic response. In particular, responders showed greater improvement in selected functional domains, including normal work and relations with other people. Although these analyses were secondary and not adjusted for multiplicity, they suggest that pain reduction was associated with measurable improvements in patient-reported functioning. Overall, these findings strengthen the clinical and translational relevance of the study and support the incorporation of multidimensional patient-reported outcomes, including pain intensity, functional interference, rescue opioid use, and quality-of-life measures, in future pharmacokinetic and pharmacogenetic studies of TDF.

This study has several limitations. First, its single-center design and relatively small sample size limit generalizability and reduce statistical power, particularly for pharmacogenetic subgroup analyses. With only 11 non-responders, the study was underpowered to detect modest genotype-phenotype associations after correction for multiple testing. Therefore, both positive and negative pharmacogenetic findings should be interpreted cautiously. Second, psychological and social determinants of pain were not prospectively assessed. Validated instruments for depression, anxiety, sleep disturbance, pain catastrophizing, social support, or existential distress were not included. This represents an important source of unmeasured confounding, particularly in a population composed almost entirely of patients with advanced disease. Third, pain mechanisms were not systematically classified using validated tools such as DN4 or LANSS. Therefore, the responder/non-responder classification may have grouped patients with nociceptive, neuropathic, and mixed pain syndromes, which may differ in opioid responsiveness. Fourth, the use of a ≥2-point reduction in NRS score as the primary definition of analgesic response represents an additional limitation. Although this criterion is pragmatic and commonly used in clinical practice and pain research, the NRS is a unidimensional measure that primarily reflects pain intensity and does not fully capture the multidimensional burden of cancer pain. Sensitivity analyses using percentage reductions in pain intensity (≥30% and ≥50%) yielded results consistent with the primary analysis. Moreover, changes in NRS scores were associated with changes in BPI scores, supporting the clinical relevance of the observed pain improvement. Nevertheless, future pharmacogenetic studies should incorporate multidimensional patient-reported outcomes, including BPI severity and interference domains, rescue opioid use, and quality-of-life measures, to provide a more comprehensive assessment of analgesic response. Finally, although serial samples were collected across the 72 h patch interval, the pharmacokinetic sampling schedule remains relatively sparse for detailed individual PK modelling. AUC was estimated using the trapezoidal rule and t_max_ was derived from observed concentration-time data; therefore, the precision of these parameters may be limited, particularly in patients with atypical absorption profiles. In addition, the palliative-care context of this cohort requires consideration of the concept of total pain, encompassing physical, psychological, social, and existential dimensions. Factors such as caregiver availability, living situation, health literacy, cultural attitudes toward opioids, previous pain experiences, and patient-clinician communication may influence pain reporting, adherence, and perceived treatment benefit. These dimensions were not captured in the present study and should be incorporated into future research on personalized opioid therapy.

## Conclusion

In conclusion, this exploratory study suggests a potential, yet unconfirmed, contribution of pharmacogenetic variability to interindividual differences in TDF PK and clinical response in cancer patients. Most associations did not remain significant after correction for multiple testing, highlighting the need for cautious interpretation. Larger, adequately powered studies are necessary to determine the clinical relevance of pharmacogenetic profiling in optimizing TDF therapy and advancing personalized pain management strategies.

## Data Availability

The data supporting the findings of this study are available from the corresponding author upon reasonable request, subject to institutional, ethical, and privacy restrictions. Individual-level clinical and genetic data are not publicly available because of privacy and ethical constraints.

## References

[1] van Den Beuken-Van Everdingen MHJ De Rijke JM Kessels AG Schouten HC Van Kleef M Patijn J. Prevalence of pain in patients with cancer: a systematic review of the past 40 years. Ann Oncol. (2007) 18:1437–49. 10.1093/annonc/mdm056 17355955

[2] Fallon M Giusti R Aielli F Hoskin P Rolke R Sharma M. Management of cancer pain in adult patients: ESMO clinical practice guidelines. Ann Oncol. (2018) 29:iv166–191. 10.1093/annonc/mdy152 30052758

[3] Ahmedzai S Brooks D. Transdermal fentanyl versus sustained-release oral morphine in cancer pain: preference, efficacy, and quality of life. J Pain Symptom Manage. (1997) 13:254–61. 10.1016/S0885-3924(97)00082-1 9185430

[4] Patocka J Wu W Oleksak P Jelinkova R Nepovimova E Spicanova L. Fentanyl and its derivatives: pain-killers or man-killers? Heliyon. (2024) 10:e28795. 10.1016/j.heliyon.2024.e28795 38644874 PMC11031787

[5] Comer SD Cahill CM. Fentanyl: receptor pharmacology, abuse potential, and implications for treatment. Neurosci Biobehav Rev. (2019) 106:49–57. 10.1016/j.neubiorev.2018.12.005 30528374 PMC7233332

[6] Larsen RH Nielsen F Sørensen JA Nielsen JB. Dermal penetration of fentanyl: inter- and intraindividual variations. Pharmacol Toxicol. (2003) 93:244–8. 10.1046/j.1600-0773.2003.pto930508.x 14629737

[7] Kuip EJM Zandvliet ML Koolen SLW Mathijssen RHJ Van Der Rijt CCD. A review of factors explaining variability in fentanyl pharmacokinetics; focus on implications for cancer patients. Br J Clin Pharmacol. (2017) 83:294–313. 10.1111/bcp.13129 27619152 PMC5237702

[8] World Health Organization. Cancer Pain Relief. Geneva: World Health Organization (1986).

[9] Kokubun H Ebinuma K Matoba M Takayanagi R Yamada Y Yago K. Population pharmacokinetics of transdermal fentanyl in patients with cancer-related pain. J Pain Palliat Care Pharmacother. (2012) 26:98–104. 10.3109/15360288.2012.679725 22764844

[10] Thompson JP Bower S Liddle AM Rowbotham DJ. Perioperative pharmacokinetics of transdermal fentanyl in elderly and young adult patients. Br J Anaesth. (1998) 81:152–4. 10.1093/bja/81.2.152 9813514

[11] Ashburn MA Ogden LL Zhang J Love G Basta SV. The pharmacokinetics of transdermal fentanyl delivered with and without controlled heat. J Pain. (2003) 4:291–7. 10.1016/S1526-5900(03)00618-7 14622685

[12] Carlini A Scarpi E Bettini C Ardizzoni A Donati CM Fabbri L. Transdermal fentanyl in patients with cachexia-A scoping review. Cancers (Basel). (2024) 16:3094. 10.3390/cancers16173094 39272951 PMC11394034

[13] Takashina Y Naito T Mino Y Yagi T Ohnishi K Kawakami J. Impact of CYP3A5 and ABCB1 gene polymorphisms on fentanyl pharmacokinetics and clinical responses in cancer patients undergoing conversion to a transdermal system. Drug Metab Pharmacokinet. (2012) 27:414–21. 10.2133/dmpk.DMPK-11-RG-134 22277678

[14] Barratt DT Bandak B Klepstad P Dale O Kaasa S Christrup LL. Genetic, pathological and physiological determinants of transdermal fentanyl pharmacokinetics in 620 cancer patients of the EPOS study. Pharmacogenet Genomics. (2014) 24:185–94. 10.1097/FPC.0000000000000032 24469018

[15] Gong X-D Wang J-Y Liu F Yuan H-H Zhang W-Y Guo Y-H. Gene polymorphisms of OPRM1 A118G and ABCB1 C3435T may influence opioid requirements in Chinese patients with cancer pain. Asian Pac J Cancer Prev. (2013) 14:2937–43. 10.7314/APJCP.2013.14.5.2937 23803057

[16] Rakvåg TT Ross JR Sato H Skorpen F Kaasa S Klepstad P. Genetic variation in the *catechol-O-methyltransferase (COMT)* gene and morphine requirements in cancer patients with pain. Mol Pain. (2008) 4:64. 10.1186/1744-8069-4-64 19094200 PMC2644687

[17] Fladvad T Klepstad P Langaas M Dale O Kaasa S Caraceni A. Variability in UDP-glucuronosyltransferase genes and morphine metabolism: observations from a cross-sectional multicenter study in advanced cancer patients with pain. Pharmacogenet Genomics. (2013) 23:117–26. 10.1097/FPC.0b013e32835ce485 23277092

[18] Montanari S Davani L Terenzi C Maltoni M Andrisano V De Simone A. Fentanyl pharmacokinetics in blood of cancer patients by gas chromatography-mass spectrometry. J Pharm Biomed Anal. (2022) 219:114913. 10.1016/j.jpba.2022.114913 35810723

[19] Chiou WL Buehler LJ. Critical evaluation of the potential error in pharmacokinetic studies of using the linear trapezoidal rule method for the calculation of the area under the plasma level-time curve. J Pharmacokinet Biopharm. (1978) 6:539–46. 10.1007/BF01062108 731416

[20] Caraceni A Hanks G Kaasa S Bennett MI Brunelli C Cherny N. Use of opioid analgesics in the treatment of cancer pain: evidence-based recommendations from the EAPC. Lancet Oncol. (2012) 13:e58–68. 10.1016/S1470-2045(12)70040-2 22300860

[21] Tassinari D Drudi F Rosati M Maltoni M. Transdermal opioids as front line treatment of moderate to severe cancer pain: a systematic review. Palliat Med. (2011) 25:478–87. 10.1177/0269216311404274 21708854

[22] Wang D-D Ma T-T Zhu H-D Peng C-B. Transdermal fentanyl for cancer pain: trial sequential analysis of 3406 patients from 35 randomized controlled trials. J Cancer Res Ther. (2018) 14:S14–21. 10.4103/0973-1482.171368 29578144

[23] Solassol I Bressolle F Caumette L Garcia F Poujol S Culine S. Inter- and intraindividual variabilities in pharmacokinetics of fentanyl after repeated 72 h transdermal applications in cancer pain patients. Ther Drug Monit. (2005) 27:491–8. 10.1097/01.ftd.0000160717.50704.42 16044107

[24] Radbruch L Sabatowski R Petzke F Brunsch-Radbruch A Grond S Lehmann KA. Transdermal fentanyl for the management of cancer pain: a survey of 1005 patients. Palliat Med. (2001) 15:309–21. 10.1191/026921601678320296 12054148

[25] Kuip E. Fentanyl pharmacokinetics: the role of clinical factors in patients with cancer using transdermal or sublingual fentanyl (Doctoral thesis). Erasmus University Rotterdam, Rotterdam, Netherlands (2019).

[26] Koolen SL Van der Rijt CC. Is there a role for pharmacogenetics in the dosing of fentanyl? Pharmacogenomics. (2017) 18:417–9. 10.2217/pgs-2017-0022 28346084

[27] Azizoddin DR Schreiber K Beck MR Enzinger AC Hruschak V Darnall BD. Chronic pain severity, impact, and opioid use among patients with cancer: an analysis of biopsychosocial factors using the CHOIR learning health care system. Cancer. (2021) 127(17):3254–63. 10.1002/cncr.33645 34061975 PMC9981278

[28] Azizoddin DR Wilson JM Flowers KM Beck M Chai P Enzinger AC. Daily pain and opioid administration in hospitalized patients with cancer: the importance of psychological factors, recent surgery, and current opioid use. Pain. (2023) 164(8):1820–7. 10.1097/j.pain.0000000000002880 36893325 PMC10363176

[29] Greenblatt DJ Von Moltke LL. Gender has a small but statistically significant effect on clearance of CYP3A substrate drugs. J Clin Pharmacol. (2008) 48:1350–5. 10.1177/0091270008323754 18757784

[30] Muscogiuri G Verde L Vetrani C Barrea L Savastano S Colao A. Obesity: a gender-view. J Endocrinol Invest. (2024) 47:299–306. 10.1007/s40618-023-02196-z 37740888 PMC10859324

[31] National Center for Biotechnology Information (NCBI). TPMT thiopurine S-methyltransferase [Homo sapiens (human)]—Gene—NCBI. Available online at: https://www.ncbi.nlm.nih.gov/gene/7172 (Accessed February 6, 2026).

[32] Mu H Ye L Wang B. Detailed resume of S-methyltransferases: categories, structures, biological functions and research advancements in related pathophysiology and pharmacotherapy. Biochem Pharmacol. (2024) 226:116361. 10.1016/j.bcp.2024.116361 38876259

[33] PubChem. UGT2B7—UDP glucuronosyltransferase family 2 member B7 (human). Available online at: https://pubchem.ncbi.nlm.nih.gov/gene/UGT2B7/human (Accessed February 6, 2026).

[34] Coffman BL King CD. The glucuronidation of opioids, other xenobiotics, and androgens by human UGT2B7Y(268) and UGT2B7H(268). Drug Metab Dispos. (1998) 26:73–7. 10.1124/dmd.26.1.73 9443856

[35] Yang Z Yin Q Li X. Influences of UGT2B7 rs7439366 and rs12233719 polymorphisms on fentanyl sensitivity in Chinese gynecologic patients. Med Sci Monit. (2020) 26:e924153-1–5. 10.12659/MSM.924153 32401749 PMC7245057

[36] Langmia IM Just KS Yamoune S Brockmöller J Masimirembwa C Stingl JC. CYP2B6 Functional variability in drug metabolism and exposure across populations—implication for drug safety, dosing, and individualized therapy. Front Genet. (2021) 12:692234. 10.3389/fgene.2021.692234 34322158 PMC8313315

[37] Andreassen TN Eftedal I Klepstad P Davies A Bjordal K Lundström S. Do CYP2D6 genotypes reflect oxycodone requirements for cancer patients treated for cancer pain? A cross-sectional multicentre study. Eur J Clin Pharmacol. (2012) 68:55–64. 10.1007/s00228-011-1093-5 21735164 PMC3249195

[38] Ziesenitz VC König SK Mahlke N Jantos R Skopp G Weiss J. Fentanyl pharmacokinetics is not dependent on hepatic uptake by organic anion-transporting polypeptide 1B1 in human beings. Basic Clin Pharmacol Toxicol. (2013) 113:43–8. 10.1111/bcpt.12066 23480028

