# Supplementary material for: Impact of genetic polymorphisms on the response to transdermal fentanyl in cancer patients—a single-center, prospective, biological study
Source: Front Pain Res (Lausanne). 2026 Aug 17;7:1834786. doi: 10.3389/fpain.2026.1834786 (PMC13527003; doi:10.3389/fpain.2026.1834786)
Supplement: Supplementary file 1 [file Datasheet1.pdf]

Supplementary Text S1.

Genomic DNA was extracted from peripheral whole blood samples using the Maxwell® RSC Whole Blood DNA Kit (Promega), according to the manufacturer's instructions. Genotyping was performed using the Axiom™ PharmacoFocus Assay (Thermo Fisher Scientific), which interrogates approximately 2,000 variants across 150 pre-defined genes involved in drug PK and pharmacodynamics. These include genes encoding drug-metabolising enzymes (e.g., CYP family), transporters (e.g., ABCB1), opioid receptors (e.g., OPRM1), and conjugating enzymes (e.g., UGT2B7 and NAT2), among others. The panel also included additional genes not directly related to fentanyl metabolism (e.g., TPMT, VKORC1), incorporated as part of the predefined assay content. Genotypic results were translated into predicted functional phenotypes according to standardised allele-function classifications. For most metabolic enzymes, metaboliser status was defined as ultrarapid, rapid, normal, intermediate, or poor. Transporter genes and MTRNR1 variants were categorised as increased, normal, decreased, or poor function. Variants in CACNA1S and RYR1 were classified as either indeterminate or associated with malignant hyperthermia susceptibility. For G6PD, the classification included indeterminate or deficient genotypes, reflecting hemolysis risk. IFNL3 polymorphisms were labeled as favorable or unfavorable response genotypes, relevant to PEG-interferon-based treatments. The NAT gene family was categorised by acetylation activity into rapid, intermediate, slow, or absent. Finally, polymorphisms in VKORC1 and CYP2C RS12777823 were classified as Resistant++, Resistant+, Normal, Sensitive-, or Sensitive--. Based on the predicted functional consequences of the genotypes, patients were further categorised into two groups: normal-function and altered-function. The normal function group included individuals with normal metabolizer (NM), intermediate acetylator (IA), or favourable response genotypes. The altered function group comprised those with intermediate or poor metabolizer status (IM, PM), decreased or poor function (DF, PF), slow or rapid acetylator phenotypes (SA, RA), resistant or sensitive genotypes, and unfavourable response genotypes.

Supplementary Figure S1. Distribution of Numeric Rating Scale (NRS) pain scores at baseline (T0) and at T4 following initiation of transdermal fentanyl. Boxplots show the median and interquartile range. Individual patient trajectories are displayed with connecting lines.

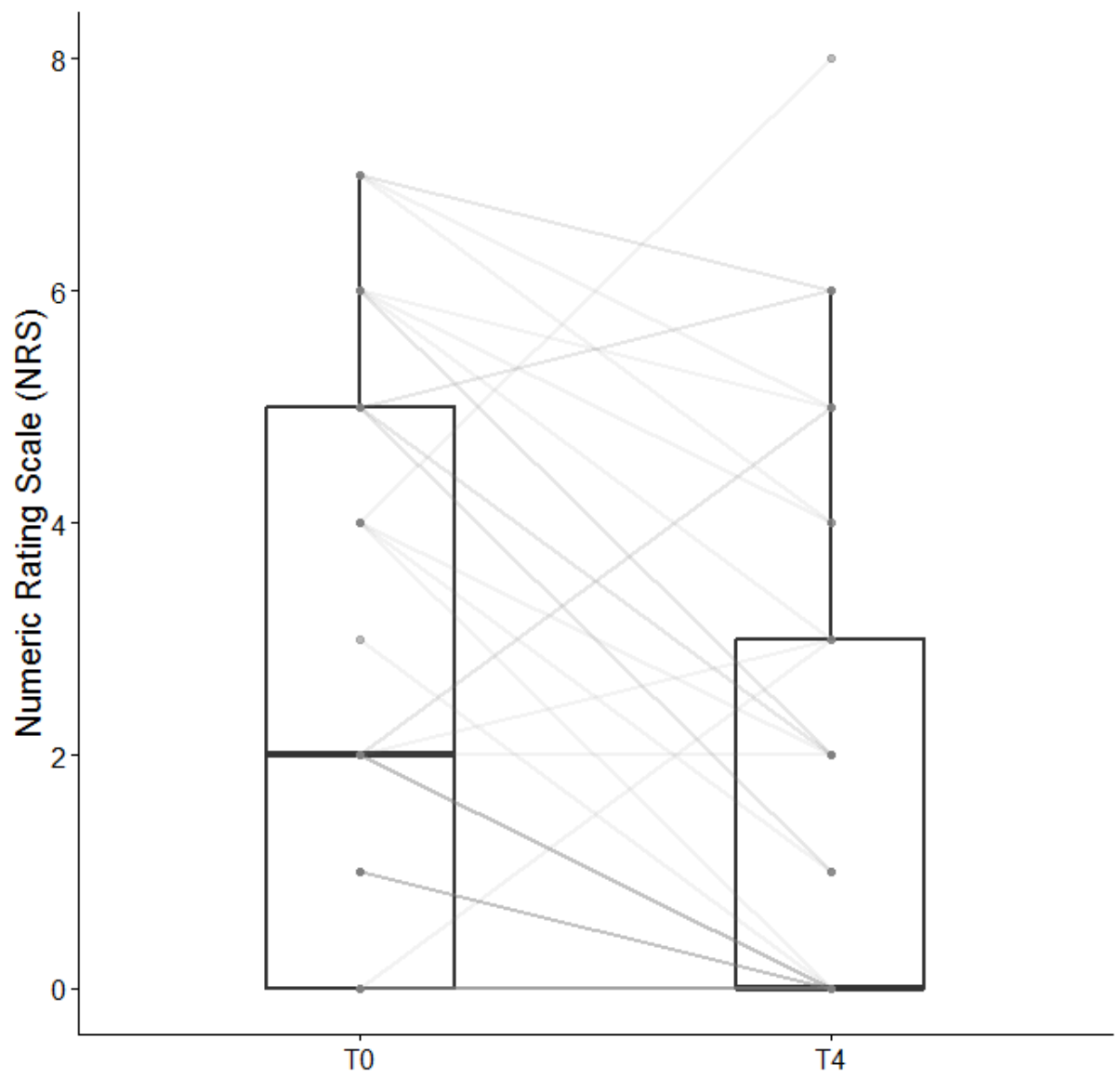

Supplementary Table S1. Genotype-to-phenotype translation and analytical grouping for pharmacogenetic analyses.

Gene/Gene group	Predicted functional phenotype categories	Normal-function/Reference group	Altered-function group
Metabolic enzymes (e.g., CYP3A4/5)	Ultrarapid metabolizer<br>Rapid metabolizer<br>Normal metabolizer<br>Intermediate metabolizer<br>Poor metabolizer	Normal metabolizer	Ultrarapid metabolizer<br>Rapid metabolizer<br>Intermediate metabolizer<br>Poor metabolizer
Transporter genes (e.g., ABCB1)	Increased function<br>Normal function<br>Decreased function<br>Poor function	Normal function	Increased function<br>Decreased function<br>Poor function
OPRM1<br>COMT<br>UGT2B7	Predicted functional categories according to allele-function classification	Normal phenotype	Altered predicted phenotype
NAT gene family	Rapid acetylator<br>Intermediate acetylator<br>Slow acetylator<br>Absent acetylator	Intermediate acetylator	Rapid acetylator<br>Slow acetylator<br>Absent acetylator
MTRNR1	Increased function<br>Normal function<br>Decreased function<br>Poor function	Normal function	Increased function<br>Decreased function<br>Poor function
CACNA1S<br>RYR1	Indeterminate malignant hyperthermia susceptibility<br>Malignant hyperthermia susceptibility	Indeterminate susceptibility phenotype	Malignant hyperthermia susceptibility phenotype
G6PD	Indeterminate genotype<br>Deficient genotype	Indeterminate phenotype	Deficient genotype

IFNL3	Favorable response genotype<br>Unfavorable response genotype	Favorable response genotype	Unfavorable response genotype
VKORC1<br>CYP2C rs12777823	Resistant++<br>Resistant+<br>Normal<br>Sensitive-<br>Sensitive--	Normal	Resistant++<br>Resistant+<br>Normal<br>Sensitive-<br>Sensitive--
TPMT	Normal metabolizer<br>Intermediate metabolizer<br>Poor metabolizer	Normal metabolizer	Intermediate metabolizer<br>Poor metabolizer

Supplementary Table S2. Concomitant medications recorded at baseline in the overall study population. Data are presented as the number and percentage of patients receiving each medication. Percentages were calculated using the overall study population as denominator (n = 49). Patients could receive more than one concomitant medication; therefore, percentages do not sum to 100%.

**Concomitant medications**	**N.**	**%**
accufil	5	10,2
acetylsalicylic acid	3	6,1
acyclovir	4	8,2
alendronate	1	2,0
allopurinol	10	20,4
alprazolam	2	4,1
amiodarone	1	2,0
amitriptyline	4	8,2
amoxicillin	3	6,1
amoxicillin + clavulanic acid	1	2,0
atorvastatin	3	6,1
bisoprolol	8	16,3
hyoscine butylbromide	1	2,0
calcium carbonate/gluconate	8	16,3
canagliflozin	1	2,0
carvedilol	1	2,0

ceftazidime	1	2,0
ceftriaxone	2	4,1
chlorpromazine	1	2,0
cholecalciferol	2	4,1
ciproxin	1	2,0
clodronate	1	2,0
clonazepam	4	8,2
clopidogrel	2	4,1
delorazepam	6	12,2
dexamethasone	23	46,9
diazepam	1	2,0
domperidone	1	2,0
duloxetine	1	2,0
dutasteride	2	4,1
enoxaparin	18	36,7
epoetin zeta	2	4,1
escitalopram	1	2,0
fenofibrate	1	2,0
fentanyl citrate	2	4,1
ferrous sulfate	2	4,1

fluconazole	4	8,2
folic acid	5	10,2
furosemide	17	34,7
haloperidol	1	2,0
hydroxyurea	1	2,0
ibuprofen	2	4,1
insulin (basal-bolus)	6	12,2
isoniazid	1	2,1
lactulose	6	12,2
levetiracetam	1	2,0
levodropropizine	1	2,0
levofloxacin	8	16,3
levosulpiride	3	6,1
levothyroxine	6	12,2
loperamide	2	4,1
lorazepam	1	2,0
lormetazepam	2	4,1
losartan	1	2,0
macrogol	19	38,8
mannitol	7	14,3

pethidine	1	2,0
metformin	1	2,0
metilprednisolone	3	6,1
metoclopramide	10	20,4
metronidazole	1	2,0
midazolam	1	2,0
mirtazapine	1	2,0
morphine hydrochloride	13	26,5
morphine sulfate controlled-release	1	2,0
morphine sulfate immediate release	8	16,3
N-acetylcysteine	3	6,1
nebivololo	1	2,0
neostigmine	1	2,0
nifedipine	1	2,0
nystatin	12	24,5
omeprazole	15	30,6
ondansetron	1	2,0
oxycodone	3	6,1
oxycodone + naloxone	4	8,2
oxycodone + paracetamol	1	2,0

pantoprazole	9	18,4
paracetamol + codeine	1	2,0
paroxetine	2	4,1
potassium canrenoate	5	10,2
potassium chloride	9	18,4
prednisone	2	4,1
pregabalin	16	32,6
rabeprazolo	1	2,0
ramipril	4	8,2
ranitidine	4	8,2
rifaximin	1	2,0
rosuvastatin	1	2,0
senna glycosides	5	10,2
sertraline	2	4,1
silodosin	1	2,0
simvastatin	2	4,1
sitagliptin	1	2,0
co-trimoxazole	7	14,3
tapentadol	1	2,0
tramadol	1	2,0

tranexamic acid	2	4,1
trazodone	1	2,0
triazolam	3	6,1
venlafaxine	1	2,0
warfarin	1	2,0
zolpidem	3	6,1

Supplementary Table S3. Pharmacokinetic parameters and Clinical Response.

	**Non-responders (n=11)**	**Responders (n=38)**	**p**
**AUC –** median (1Q-3Q)	252 (81.2-440)	149 (56.9-873)	0.75
$\mathbf{T_{max}}$ – median (1Q-3Q)	3 (1.5-3.5)	3 (1-3)	0.61
$\mathbf{C_{max}}$ – median (1Q-3Q)	4.7 (1.31-13.4)	4 (1.43-17.1)	0.92

Abbreviations: 1Q, First Quartile; 3Q, Third Quartile

Supplementary Table S4. Genetic Polymorphisms and Clinical Response.

Gene	Phenotype	Non-responders (n=11)	Responders (n=38)	p	padj
CYP2CRS12777823	Normal function	10	26		
CYP2CRS12777823	Altered function	1	12	0.25	1
IFNL3	Normal function	7	20		
IFNL3	Altered function	4	18	0.73	1
VKORC1	Normal function	1	2		
VKORC1	Altered function	8	19		
VKORC1	NA	2	17	1	1
NAT1	Normal function	0	2		
NAT1	Altered function	11	36	1	1
NAT2	Normal function	3	11		
NAT2	Altered function	3	20		
NAT2	NA	5	7	0.65	1
COMT	Normal function	4	10		
COMT	Altered function	7	27		
COMT	NA	0	1	0.71	1
CYP1A2	Normal function	11	36		
CYP1A2	Altered function	0	2	1	1
CYP2A6	Normal function	8	28		
CYP2A6	Altered function	2	8		
CYP2A6	NA	1	2	1	1
CYP2B6	Normal function	5	17		
CYP2B6	Altered function	6	17		
CYP2B6	NA	0	4	1	1
CYP2C8	Normal function	9	23		
CYP2C8	Altered function	1	13		
CYP2C8	NA	1	2	0.14	1
CYP2C9	Normal function	9	23		
CYP2C9	Altered function	2	14		
CYP2C9	NA	0	1	0.29	1
CYP2C19	Normal function	4	15		
CYP2C19	Altered function	7	22		
CYP2C19	NA	0	1	1	1
CYP2D6	Normal function	8	21		
CYP2D6	Altered function	3	14		

CYP2D6	NA	0	3	0.50	1
CYP2E1	Normal function	10	27		
CYP2E1	Altered function	1	10		
CYP2E1	NA	0	1	0.42	1
CYP3A4	Normal function	10	33		
CYP3A4	Altered function	1	5	1	1
CYP3A5	Normal function	0	1		
CYP3A5	Altered function	11	37	1	1
CYP3A7	Normal function	9	29		
CYP3A7	Altered function	2	9	1	1
CYP4F2	Normal function	3	15		
CYP4F2	Altered function	8	23	0.72	1
DPYD	Normal function	11	37		
DPYD	Altered function	0	1	1	1
GSTM1	Normal function	0	6		
GSTM1	Altered function	11	32	0.31	1
GSTP1	Normal function	4	15		
GSTP1	Altered function	7	23	1	1
NUDT15	Normal function	10	37		
NUDT15	Altered function	1	1	0.40	1
TPMT	Normal function	8	38		
TPMT	Altered function	3	0	0.009	0.22
UGT1A1	Normal function	5	19		
UGT1A1	Altered function	6	19	1	1
UGT2B7	Normal function	4	13		
UGT2B7	Altered function	6	22		
UGT2B7	NA	1	3	1	1

Abbreviations: NA, Not Available.

Supplementary Table S5. Genetic Polymorphisms and Dose Category.

Gene	Phenotype	High dose (n=12)	Low dose (n=37)	p	padj
CYP2CRS12777823	Normal function	9	27		
CYP2CRS12777823	Altered function	3	10	1	1
IFNL3	Normal function	7	20		
IFNL3	Altered function	5	17	1	1
VKORC1	Normal function	0	3		
VKORC1	Altered function	7	20		
VKORC1	NA	5	14	1	1
NAT1	Normal function	1	1		
NAT1	Altered function	11	36	0.43	1
NAT2	Normal function	2	12		
NAT2	Altered function	7	16		
NAT2	NA	3	9	0.43	1
COMT	Normal function	5	9		

COMT	Altered function	6	28		
COMT	NA	1	0	0.26	1
CYP1A2	Normal function	12	35		
CYP1A2	Altered function	0	2	1	1
CYP2A6	Normal function	8	28		
CYP2A6	Altered function	3	7		
CYP2A6	NA	1	2	0.68	1
CYP2B6	Normal function	4	18		
CYP2B6	Altered function	7	16		
CYP2B6	NA	1	3	0.49	1
CYP2C8	Normal function	9	23		
CYP2C8	Altered function	3	11		
CYP2C8	NA	0	3	0.73	1
CYP2C9	Normal function	6	26		
CYP2C9	Altered function	5	11		
CYP2C9	NA	1	0	0.47	1
CYP2C19	Normal function	5	14		
CYP2C19	Altered function	7	22		
CYP2C19	NA	0	1	1	1
CYP2D6	Normal function	7	22		
CYP2D6	Altered function	4	13		
CYP2D6	NA	1	2	1	1
CYP2E1	Normal function	8	29		
CYP2E1	Altered function	4	7		
CYP2E1	NA	0	1	0.43	1
CYP3A4	Normal function	11	32		
CYP3A4	Altered function	1	5	1	1
CYP3A5	Normal function	0	1		
CYP3A5	Altered function	12	36	1	1
CYP3A7	Normal function	9	29		
CYP3A7	Altered function	3	8	1	1
CYP4F2	Normal function	4	14		
CYP4F2	Altered function	8	23	1	1
DPYD	Normal function	12	36		
DPYD	Altered function	0	1	1	1
GSTM1	Normal function	2	4		
GSTM1	Altered function	10	33	0.63	1
GSTP1	Normal function	7	12		
GSTP1	Altered function	5	25	0.17	1
NUDT15	Normal function	11	36		
NUDT15	Altered function	1	1	0.43	1
TPMT	Normal function	12	34		
TPMT	Altered function	0	3	0.57	1
UGT1A1	Normal function	6	18		
UGT1A1	Altered function	6	19	1	1
UGT2B7	Normal function	3	14		

UGT2B7	Altered function	9	19		
UGT2B7	NA	0	4	0.49	1

Abbreviations: NA, Not Available.

Supplementary Table S6. Genetic Polymorphisms and Side Effects.

Gene	Phenotype	No side effects (n=30)	Side effects (n=16)	p	padj
CYP2CRS12777823	Normal function	22	11		
CYP2CRS12777823	Altered function	8	5	0.74	1
IFNL3	Normal function	18	7		
IFNL3	Altered function	12	9	0.36	1
VKORC1	Normal function	2	1		
VKORC1	Altered function	19	5		
VKORC1	NA	9	10	0.55	1
NAT1	Normal function	1	1		
NAT1	Altered function	29	15	1	1
NAT2	Normal function	7	5		
NAT2	Altered function	13	9		
NAT2	NA	10	2	1	1
COMT	Normal function	8	5		
COMT	Altered function	21	11		
COMT	NA	1	0	1	1
CYP1A2	Normal function	29	15		
CYP1A2	Altered function	1	1	1	1
CYP2A6	Normal function	24	10		
CYP2A6	Altered function	6	4		
CYP2A6	NA	0	2	0.70	1
CYP2B6	Normal function	14	6		
CYP2B6	Altered function	12	10		
CYP2B6	NA	4	0	0.35	1
CYP2C8	Normal function	19	10		
CYP2C8	Altered function	9	5		
CYP2C8	NA	2	1	1	1
CYP2C9	Normal function	22	9		
CYP2C9	Altered function	8	6		
CYP2C9	NA	0	1	0.50	1
CYP2C19	Normal function	12	6		
CYP2C19	Altered function	17	10		
CYP2C19	NA	1	0	1	1
CYP2D6	Normal function	18	9		
CYP2D6	Altered function	10	6		
CYP2D6	NA	2	1	1	1
CYP2E1	Normal function	24	11		
CYP2E1	Altered function	5	5		
CYP2E1	NA	1	0	0.46	1

CYP3A4	Normal function	25	15		
CYP3A4	Altered function	5	1	0.65	1
CYP3A5	Normal function	1	0		
CYP3A5	Altered function	29	16	1	1
CYP3A7	Normal function	26	11		
CYP3A7	Altered function	4	5	0.24	1
CYP4F2	Normal function	8	8		
CYP4F2	Altered function	22	8	0.19	1
DPYD	Normal function	30	15		
DPYD	Altered function	0	1	0.35	1
GSTM1	Normal function	4	1		
GSTM1	Altered function	26	15	0.64	1
GSTP1	Normal function	11	5		
GSTP1	Altered function	19	11	0.76	1
NUDT15	Normal function	29	16		
NUDT15	Altered function	1	0	1	1
TPMT	Normal function	28	15		
TPMT	Altered function	2	1	1	1
UGT1A1	Normal function	15	8		
UGT1A1	Altered function	15	8	1	1
UGT2B7	Normal function	9	8		
UGT2B7	Altered function	18	7		
UGT2B7	NA	3	1	0.33	1

Abbreviations: NA, Not Available.

Supplementary Table S7. Genetic Polymorphisms and Pharmacokinetic Parameters.

		AUC			T_{max}			C_{max}		
**Gene**	**Phenotype**	**median (1Q-3Q)**	**p**	**padj**	**median (1Q-3Q)**	**p**	**padj**	**median (1Q-3Q)**	**p**	**padj**
CYP2CRS12777823	Normal function	148.95 (58.02–547.11)			3.00 (1.00–3.50)			2.65 (1.26–13.41)		
CYP2CRS12777823	Altered function	187.06 (84.89–1083.64)	0.80	0.93	3.00 (1.00–3.00)	0.89	0.92	4.14 (1.53–17.13)	1	1
IFNL3	Normal function	231.17 (59.96–786.83)			3.00 (1.00–3.00)			4.70 (1.26–18.22)		
IFNL3	Altered function	108.32 (56.91–305.55)	0.41	0.93	3.00 (1.00–4.00)	0.43	0.92	2.38 (1.43–6.70)	0.45	0.89
VKORC1	Normal function	108.32 (93.25–496.72)			3.00 (2.00–3.00)			1.98 (1.70–9.59)		
VKORC1	Altered function	164.78 (58.85–965.64)	1	1	3.00 (1.00–3.00)	1	1	4.23 (1.30–19.80)	0.85	0.89
NAT1	Normal function	76.36 (76.36–76.36)			3.00 (3.00–3.00)			1.51 (1.51–1.51)		
NAT1	Altered function	164.78 (58.02–786.83)	0.67	0.93	3.00 (1.00–3.50)	0.79	0.92	4.14 (1.26–17.04)	0.67	0.89
NAT2	Normal function	210.14 (90.88–1296.83)			3.00 (1.25–4.00)			4.80 (1.80–29.55)		
NAT2	Altered function	73.32 (53.30–279.13)	0.14	0.61	2.50 (1.00–3.00)	0.26	0.92	1.47 (1.00–6.09)	0.09	0.78
COMT	Normal function	272.54 (90.75–1193.85)			3.00 (2.00–3.00)			4.61 (1.96–24.58)		
COMT	Altered function	134.44 (52.80–326.85)	0.10	0.61	3.00 (1.00–4.00)	0.88	0.92	4.00 (1.08–11.90)	0.19	0.78
CYP1A2	Normal function	156.86 (57.46–829.90)			3.00 (1.00–3.75)			4.07 (1.18–17.08)		
CYP1A2	Altered function	153.76 (115.06–192.47)	0.82	0.93	3.00 (3.00–3.00)	0.69	0.92	3.13 (2.32–3.94)	0.86	0.89
CYP2A6	Normal function	164.78 (59.96–792.91)			3.00 (1.00–4.00)			4.23 (1.43–17.07)		
CYP2A6	Altered function	160.75 (60.83–378.82)	0.70	0.93	3.00 (3.00–3.00)	0.73	0.92	4.07 (1.31–10.57)	0.76	0.89
CYP2B6	Normal function	148.95 (42.35–553.98)			2.00 (1.00–3.00)			2.65 (0.83–14.91)		
CYP2B6	Altered function	135.70 (64.63–984.54)	0.39	0.93	3.00 (2.00–4.00)	0.10	0.85	4.00 (1.47–17.07)	0.44	0.89
CYP2C8	Normal function	164.78 (58.02–627.33)			3.00 (1.00–3.00)			4.23 (1.09–16.02)		
CYP2C8	Altered function	126.11 (62.01–1083.87)	0.89	0.93	3.00 (1.00–4.00)	0.45	0.92	2.52 (1.46–18.72)	0.82	0.89
CYP2C9	Normal function	135.70 (58.85–627.33)			3.00 (1.00–3.00)			2.50 (1.26–16.05)		
CYP2C9	Altered function	190.06 (58.47–623.42)	0.89	0.93	3.00 (1.75–3.25)	0.33	0.92	4.38 (1.33–11.95)	0.71	0.89
CYP2C19	Normal function	164.78 (67.32–712.68)			3.00 (1.00–3.00)			4.23 (1.43–13.71)		
CYP2C19	Altered function	161.38 (60.38–743.76)	0.86	0.93	3.00 (1.00–4.00)	0.78	0.92	4.07 (1.40–16.99)	0.82	0.89
CYP2D6	Normal function	231.17 (68.47–872.97)			3.00 (1.00–3.00)			4.70 (1.51–17.13)		
CYP2D6	Altered function	135.70 (56.46–396.14)	0.50	0.93	3.00 (2.00–4.00)	0.17	0.92	2.65 (1.43–6.57)	0.60	0.89

CYP2E1	Normal function	142.32 (56.04–590.65)			3.00 (1.00–3.00)			3.33 (1.08–15.46)		
CYP2E1	Altered function	187.06 (72.41–811.94)	0.63	0.93	3.00 (2.00–4.00)	0.40	0.92	4.14 (1.51–13.58)	0.79	0.89
CYP3A4	Normal function	187.06 (68.57–879.05)			3.00 (1.00–4.00)			4.23 (1.48–17.04)		
CYP3A4	Altered function	56.91 (54.77–68.47)	0.17	0.61	1.00 (1.00–2.00)	0.06	0.85	1.08 (0.99–1.51)	0.21	0.78
CYP3A5	Normal function	11.94 (11.94–11.94)			4.00 (4.00–4.00)			0.83 (0.83–0.83)		
CYP3A5	Altered function	164.78 (59.96–786.83)	0.08	0.61	3.00 (1.00–3.00)	0.19	0.92	4.14 (1.43–17.04)	0.25	0.78
CYP3A7	Normal function	187.06 (68.47–872.97)			3.00 (1.00–4.00)			4.23 (1.51–17.19)		
CYP3A7	Altered function	84.89 (30.53–376.93)	0.17	0.61	2.00 (1.00–3.00)	0.33	0.92	1.96 (0.92–9.57)	0.22	0.78
CYP4F2	Normal function	142.32 (57.88–1031.21)			3.00 (1.25–3.00)			2.58 (1.46–17.08)		
CYP4F2	Altered function	175.92 (63.90–486.89)	0.71	0.93	3.00 (1.00–3.75)	0.88	0.92	4.19 (1.11–11.48)	0.71	0.89
DPYD	Normal function	164.78 (59.96–786.83)			3.00 (1.00–3.50)			4.14 (1.43–17.04)		
DPYD	Altered function	56.91 (56.91–56.91)	0.5	0.93	3.00 (3.00–3.00)	0.79	0.92	1.08 (1.08–1.08)	0.46	0.89
GSTM1	Normal function	56.46 (49.95–199.88)			2.00 (1.00–3.00)			1.44 (0.99–4.23)		
GSTM1	Altered function	164.78 (64.63–786.83)	0.36	0.93	3.00 (1.00–3.50)	0.66	0.92	4.14 (1.47–17.04)	0.41	0.89
GSTP1	Normal function	105.79 (63.44–789.79)			3.00 (1.00–3.00)			2.18 (1.12–15.40)		
GSTP1	Altered function	215.53 (57.88–664.01)	0.70	0.93	3.00 (1.25–3.75)	0.54	0.92	4.47 (1.43–16.44)	0.67	0.89
NUDT15	Normal function	142.32 (57.46–660.58)			3.00 (1.00–3.00)			3.33 (1.18–15.69)		
NUDT15	Altered function	1465.55 (1009.76–1921.34)	0.10	0.61	2.50 (1.75–3.25)	0.87	0.92	27.94 (21.43–34.46)	0.10	0.78
TPMT	Normal function	135.70 (56.91–700.69)			3.00 (1.00–3.00)			2.65 (1.10–16.95)		
TPMT	Altered function	326.85 (291.18–605.99)	0.28	0.88	4.00 (3.50–4.00)	0.08	0.85	11.90 (8.63–14.55)	0.25	0.78
UGT1A1	Normal function	199.88 (77.27–513.77)			3.00 (1.00–4.00)			4.23 (1.47–14.51)		
UGT1A1	Altered function	103.27 (56.46–1083.64)	0.82	0.93	3.00 (1.00–3.00)	0.77	0.92	2.38 (1.10–16.95)	0.84	0.89
UGT2B7	Normal function	78.18 (42.35–187.06)			3.00 (1.00–4.00)			1.51 (0.83–4.14)		
UGT2B7	Altered function	326.85 (96.60–1057.86)	0.02	0.56	3.00 (1.00–3.50)	0.46	0.92	6.70 (1.97–19.80)	0.006	0.14

Abbreviations: 1Q, First Quartile; 3Q, Third Quartile.

Table 8a. Genetic Polymorphisms and Clinical Response.

**Gene phenotype – no (%)**	**Responders[1] (n=25)**	**Non responders (n=24)**	***p***	**Responders[2] (n=23)**	**Non responders (n=26)**	***p***
**CYP2CRS12777823**						
Normal function	17 (68)	19 (79)		17 (74)	19 (73)	
Altered function	8 (32)	5 (21)	0.52	6 (26)	7 (27	1.00
**CYP2C8**						
Normal function	17 (71)	15 (68)		16 (73)	16 (67)	
Altered function	7 (29)	7 (32)		6 (27	8 (33)	
Missing	1	2	1.00	1	2	0.75
**CYP2C9**						
Normal function	15 (62)	17 (71)		15 (65)	17 (68)	
Altered function	9 (38)	7 (29)		8 (35)	8 (32)	
Missing	1	0	0.76	0	1	1.00
**TPMT**						
Normal function	25 (100)	21(87)		23 (100)	23 (88)	
Altered function	0	3 (13)	0.11	0	3 (12)	0.24

[1] ≥30% reduction; [2] ≥50% reduction.

Supplementary Table S8b. Pharmacokinetic parameters and Clinical Response.

	Non-responders (n=24)	Responders[1] (n=25)	p	Non-responders (n=26)	Responders[2] (n=23)	p
**AUC –** median (1Q-3Q)	149 (76-1083)	187 (42-701)	0.41	165 (79-1084)	85 (37-540)	0.16
T_{max} – median (1Q-3Q)	3 (1-4)	2 (1-3)	0.24	3 (1-4)	2 (1-3)	0.21
C_{max} – median (1Q-3Q)	4.0 (1.5-19.3)	4.1 (1.0-16.9)	0.46	4.2 (1.5-17.1)	2.0 (0.8-10.2)	0.22

[1]≥30% reduction; [2]≥50% reduction.
Abbreviations: 1Q, First Quartile; 3Q, Third Quartile

Supplementary Table S8c. Genetic Polymorphisms and Clinical Response.

Gene	Phenotype	Non-responders (n=24)	Responders (n=25)[1]	p	Non-responders (n=26)	Responders (n=23)[2]	p
CYP2CRS12777823	Normal function	19	17		19	17	
CYP2CRS12777823	Altered function	5	8	0.52	7	6	0.95
IFNL3	Normal function	13	14		14	13	
IFNL3	Altered function	11	11	1.00	12	10	0.85
VKORC1	Normal function	2	1		2	1	
VKORC1	Altered function	14	13		14	13	
VKORC1	NA	8	11	1.00	10	9	1.00
NAT1	Normal function	1	1		1	1	
NAT1	Altered function	23	24	1.00	25	22	1.00
NAT2	Normal function	8	6		8	6	
NAT2	Altered function	8	15		10	13	
NAT2	NA	8	4	0.31	8	4	0.51
COMT	Normal function	8	6		9	5	
COMT	Altered function	16	18		17	17	
COMT	NA	0	1	0.75	0	1	0.53
CYP1A2	Normal function	23	24		25	22	
CYP1A2	Altered function	1	1	1.00	1	1	1.00
CYP2A6	Normal function	17	19		18	18	
CYP2A6	Altered function	6	4		6	4	
CYP2A6	NA	1	2	0.72	2	1	0.73
CYP2B6	Normal function	9	13		10	12	
CYP2B6	Altered function	13	10		14	9	
CYP2B6	NA	2	2	0.38	2	2	0.38
CYP2C8	Normal function	15	17		16	16	
CYP2C8	Altered function	7	7		8	6	
CYP2C8	NA	2	1	1.00	2	1	0.75
CYP2C9	Normal function	17	15		17	15	
CYP2C9	Altered function	7	9		8	8	

CYP2C9	NA	0	1	0.76	1	0	1.00
CYP2C19	Normal function	11	8		11	8	
CYP2C19	Altered function	13	16		15	12	
CYP2C19	NA	0	1	0.56	0	1	1.00
CYP2D6	Normal function	16	13		18	11	
CYP2D6	Altered function	8	9		8	9	
CYP2D6	NA	0	3	0.76	0	3	0.37
CYP2E1	Normal function	20	17		21	16	
CYP2E1	Altered function	4	7		5	6	
CYP2E1	NA	0	1	0.49	0	1	0.73
CYP3A4	Normal function	22	21		24	19	
CYP3A4	Altered function	2	4	0.67	2	4	0.40
CYP3A5	Normal function	0	1		0	1	
CYP3A5	Altered function	24	24	1.00	26	22	0.47
CYP3A7	Normal function	21	17		22	16	
CYP3A7	Altered function	3	8	0.17	4	7	0.31
CYP4F2	Normal function	7	11		9	9	
CYP4F2	Altered function	17	14	0.38	17	14	0.77
DPYD	Normal function	24	24		26	22	
DPYD	Altered function	0	1	1.00	0	1	0.47
GSTM1	Normal function	3	3		3	3	
GSTM1	Altered function	21	22	1.00	23	20	1.00
GSTP1	Normal function	12	7		13	6	
GSTP1	Altered function	12	18	0.15	13	17	0.14
NUDT15	Normal function	22	25		24	23	
NUDT15	Altered function	2	0	0.23	2	0	0.49
TPMT	Normal function	21	25		23	23	
TPMT	Altered function	3	0	0.11	3	0	0.24
UGT1A1	Normal function	11	13		12	12	
UGT1A1	Altered function	13	12	0.78	14	11	0.78
UGT2B7	Normal function	7	10		8	9	

UGT2B7	Altered function	14	14		15	13	
UGT2B7	NA	3	1	0.76	3	1	0.76

[1] 2-point reduction; [2] ≥30% reduction; [3] ≥50% reduction.

Abbreviations: NA, Not Available.

Table S9a. Genetic Polymorphisms and Clinical Response excluding haematological malignancies.

Gene phenotype – no (%)	Responders[1] (n=35)	Non responders (n=8)	*p*	*p*-adj	Responders[2] (n=23)	Non responders (n=20)	*p*	*p*-adj	Responders[3] (n=22)	Non responders (n=21)	*p*	*p*-adj
**CYP2CRS12777823**												
Normal function	24 (69)	7 (87)			16 (70)	15 (75)			16 (73)	15 (71)		
Altered function	11 (31)	1 (13)	0.41	0.76	7 (30)	5 (25)	0.74	0.96	6 (27)	6 (29)	1.00	1.00
**CYP2C8**												
Normal function	21 (64)	6 (86)			16 (73)	11 (61)			15 (71)	12 (63)		
Altered function	12 (36)	1 (14)			6 (27)	7 (39)			6 (29)	7 (37)		
Missing	2	1	0.39	0.76	1	2	0.51	0.76	1	2	0.74	0.96
**CYP2C9**												
Normal function	21 (60)	6 (75)			14 (61)	13 (65)			14 (64)	13 (62)		
Altered function	14 (40)	2 (25)			9 (39)	7 (35)			8 (36)	8 (38)		
Missing	0	0	0.69	0.96	0	0	1.00	1.00	0	0	1.00	1.00
**TPMT**												
Normal function	35 (100)	5 (62)			23 (100)	17 (85)			22 (100)	18 (86)		
Altered function	0	3 (38)	0.004	0.07	0	3 (15)	0.09	0.45	0	3 (14)	0.11	0.45

[1] 2-point reduction; [2] ≥30% reduction; [3] ≥50% reduction.

Table S9b. Genetic Polymorphisms and Pharmacokinetics excluding haematological malignancies..

**Gene phenotype**	**AUC**			**T_{max}**			**C_{max}**		
	median, 1Q-3Q	***p***	***p*-adj**	median, 1Q-3Q	***p***	***p*-adj**	median, 1Q-3Q	***p***	***p*-adj**
**COMT**									
Normal function	396.14 (84.89-1230.59)			3.00 (2.00-3.00)			6.57 (1.96-26.34)		
Altered function	156.86 (56.46-326.85)	0.13	0.51	3.00 (1.00-4.00)	1.00	1.00	4.07 (1.08-11.90)	0.21	0.57
**CYP3A4**									
Normal function	187.06 (78.18-553.98)			3.00 (1.00-4.00)			4.23 (1.53-14.91)		
Altered function	68.47 (56.91-700.69)	0.61	0.96	2.00 (1.00-3.00)	0.24	0.57	1.51 (1.08-17.19)	0.70	0.96
**CYP3A5**									
Normal function	11.94 (11.94-11.94)			4.0 (4.00-4.00)			0.83 (0.83-0.83)		
Altered function	187.06 (68.47-700.69)	0.12	0.51	3.00 (1.00-3.00)	0.20	0.57	4.23 (1.51-17.13)	0.19	0.57
**NUDT15**									
Normal function	156.86 (59.96-620.46)			3.00 (1.00-3.00)			4.07 (1.43-14.51)		
Altered function	1465.55 (553.98-2377.13)	0.09	0.45	2.00 (1.00-4.00)	0.88	0.96	27.94 (14.91-40.98)	0.09	0.45
**TPMT**									
Normal function	164.78 (59.14-872.97)			3.00 (1.00-3.00)			4.14 (1.43-17.19)		
Altered function	255.51 (148.95-326.85)	0.66	0.96	4.00 (4.00-4.00)	0.02	0.17	5.37 (2.65-11.90)	0.63	0.96
**UGT2B7**									
Normal function	78.18 (37.38-187.06)			3.00 (1.00-3.00)			1.44 (0.83-4.14)		
Altered function	326.85 (134.44-1230.59)	**0.007**	0.08	3.00 (1.00-4.00)	0.85	0.96	10.22 (2.65-20.29)	**0.002**	0.07

Abbreviations: 1Q, First Quartile; 3Q, Third Quartile.

Supplementary Table S9c. Pharmacokinetic parameters and Clinical Response excluding haematological malignancies.

	Non-responders (n=8)	Responders[1] (n=35)	p	Non-responders (n=20)	Responders[2] (n=23)	p	Non-responders (n=21)	Responders[3] (n=22)	p
**AUC –** median (1Q-3Q)	200 (119-291)	176 (57-885)	0.92	165 (103-1084)	187 (37-701)	0.32	208 (106-978)	136 (37-540)	0.20
$\mathbf{T_{max}}$ – median (1Q-3Q)	3 (1-4)	3 (1-3)	0.25	3 (1-4)	2 (1-3)	0.20	3 (1-4)	2 (1-3)	0.33
$\mathbf{C_{max}}$ – median (1Q-3Q)	3.7 (2.0-8.6)	4.2 (1.4-19.3)	0.91	4.2 (2.0-19.3)	4.1 (1.0-17.1)	0.36	4.5 (2.2-18.2)	3.0 (1.0-10.2)	0.24

[1] 2-point reduction; [2] ≥30% reduction; [3] ≥50% reduction.
Abbreviations: 1Q, First Quartile; 3Q, Third Quartile.

Table S10. Correlation between changes in pain intensity and changes in BPI interference scores from T0 to T4.

	BPI Total Pain Interference		General activity		Mood		Walking ability		Work		Relationships		Sleep		Enjoyment	
	r_s	p	r_s	p	r_s	p	r_s	p	r_s	p	r_s	p	r_s	p	r_s	p
NRS change	0.39	0.006	0.15	0.288	0.08	0.586	0.13	0.367	0.26	0.066	0.45	0.001	0.08	0.598	0.14	0.338

Table S11. Association of the change in NRS scores and the change in the BPI interference domain from T0 to T4.

	**Responders (n=38)**	**Non responders (n=11)**	
**Interference domain (%)**	**Mean change (95% CI)**	**Mean change (95% CI)**	***p***
General activity	-0.58 (-1.26 to 0.10)	0.55 (-1.36 to 2.45)	0.06
Mood	-0.74 (-1.42 to -0.05)	-0.09 (-1.83 to 1.65)	0.33
Walking ability	-0.92 (-1.69 to -0.15)	0.55 (-1.05 to 2.15)	0.21
Normal work	-0.34 (-1.33 to 0.65)	1.36 (-0.02 to 2.75)	**0.008**
Relations with other people	-0.68 (-1.53 to 0.16)	1.09 (-0.51 to 2.69)	**0.01**
Sleep	-0.11 (-0.84 to 0.63)	0.64 (-1.94 to 3.21)	0.38
Enjoyment of life	-0.92 (-1.85 to 0.01)	0.30 (-2.36 to 2.96)	0.22
Pain interference score	*-4.29 (-8.44 to -0.14)*	*4.80 (-5.88 to 15.48)*	***0.02***

Abbreviations: CI, confidence interval.
